# Supplementary material for: The Role of Psychobiotics to Ensure Mental Health during the COVID-19 Pandemic—A Current State of Knowledge
Source: Int J Environ Res Public Health. 2022 Sep 3;19(17):11022. doi: 10.3390/ijerph191711022 (PMC9518511; doi:10.3390/ijerph191711022)
Supplement: Supplementary file 1 [file ijerph-19-11022-s001.zip › ijerph-1862243-supplementary.pdf]

Supplementary Materials

Table S1. Summary of registered interventional clinical trials of probiotics dietary supplementation for the prevention or treatment of COVID-19 (*via* https://www.clinicaltrials.gov/, accessed July 2022)

Clinical Trials identifier	Status	Location	Official title	Evaluation	Hypothesis	Intervention	Participants	Reference
NCT04399252	Completed	Durham, United States	A randomized trial of the effect of *Lactobacillus* on the microbiome of household contacts exposed to COVID-19	Randomized, double-blind, placebo-controlled study	Taking LGG as a probiotic will protect against COVID-19 infection and reduce the severity of disease in those who become infected, and will be associated with beneficial changes in the composition of the gut microbiome	2 capsules per day of either LGG and placebo	182	Tang et al., 2021 [1] https://clinicaltrials.gov/ct2/show/NCT04399252
NCT04390477	Completed	Alicante, Spain	The intestinal microbiota as a therapeutic target in hospitalized patients with COVID-19 infection	A prospective randomized case-control pilot study	Positive effect of probiotic on the gut microbiome that could led to produce a less severe clinical	Oral daily capsule containing $1x10^9$ CFU of the probiotic with maltodextrin as excipient,	41	https://clinicaltrials.gov/ct2/show/NCT04390477

					evolution of the disease	administrated for 30 days and placebo		
NCT04621071	Completed	Sherbrooke, Canada	Evaluation of the efficacy of probiotics to reduce the duration and symptoms of COVID-19 (PROVID-19 study)	Randomized, double-blind, controlled trial	Efficacy of probiotics to reduce the duration and symptoms of COVID-19 in a symptomatic population tested positive to SARS-CoV-2, self-caring at home	Probiotics (2 strains $10x10^9$ CFU) or placebo (potato starch and magnesium stearate) up to 25 days	17	https://clinicaltrials.gov/ct2/show/NCT04621071
NCT04847349	Completed	New Brunswick, United States	Live microbials to boost anti-SARS-CoV-2 immunity clinical trial (Live BASIC Trial)	Pilot double-blind randomized controlled trial	Two doses of a combination of live microbials (probiotics) boost the immunity of unvaccinated persons previously infected with SARS-CoV-2	Combination of a standard (or high) dose of live microbials (probiotics) taken as a capsule once per day with breakfast for 21 days and placebo	54	https://clinicaltrials.gov/ct2/show/NCT04847349
NCT04734886	Completed	Örebro, Sweden	Exploratory study on the effects of probiotic supplementation on SARS-	Randomized clinical trial	Well-known probiotic strain *L. reuteri* DSM 17938 enhance SARS-CoV-2	$1 * 10^8$ CFU of *L. reuteri* DSM 17938 + 10 ug vitamin D3, two capsules per day for 6	161	https://clinicaltrials.gov/ct2/show/NCT04734886

			CoV-2 antibody response in healthy adults		specific antibody response upon and after infection in healthy adults	months and placebo + 10 ug vitamin D3 two capsules per day over six weeks		
NCT04517422	Completed	Mexico City, Mexico	Efficacy and safety of *Lactobacillus plantarum* and *Pediococcus acidilactici* as co-adjuvant therapy for reducing the risk of severe disease in adults with SARS-CoV-2 and its modulation of the fecal microbiota	Randomized clinical trial	Combination of probiotics reduce the risk to progress to moderate or severe COVID and associated advantages such as reduce the risk of death	Combination of *Lactobacillus plantarum* CECT30292, *Lactobacillus plantarum* CECT 7484, *Lactobacillus plantarum* CECT 7485, and *Pediococcus acidilactici* CECT 7483 and placebo *Dosage not provided*	300	https://clinicaltrials.gov/ct2/show/NCT04517422
NCT05194033	Completed	Beijing, China	Efficacy and safety of oral *Lactobacillus plantarum* GUANKE (CGMCC NO.21720) in enhancement of antibody level after	Prospective, Randomized, Double-blind, Placebo-Controlled Trial (Trial 1)	Oral probiotics in SARS-CoV-2 serum neutralize antibody titer level and T cell response level	Oral probiotics 1 time/day for 7 consecutive days *Dosage not provided*	31	https://clinicaltrials.gov/ct2/show/NCT05194033

			SARS-CoV-2 vaccination					
NCT04937556	Completed	Madrid, Spain	Evaluate the effect of the *Lactobacillus* probiotic strain in the immune response in participants positive for SARS-CoV-2 infection	Randomized, double-blind, placebo-controlled study	Probiotic supplementation enhance the immune response of patients with COVID-19	A mixture of $1*10^9$ colony forming unit (CFU) of *Lactobacillus salivarius* + Vit D + Zinc citrate in 1 capsule daily administrated during 28 days and placebo	41	https://clinicaltrials.gov/ct2/show/NCT04937556
NCT05043376	Completed	Lahore, Pakistan	Study to investigate the treatment effect of probiotic *Streptococcus salivarius* K12 in hospitalized patients (non-ICU) with COVID-19	Randomized, open-label and controlled clinical trial	The lung presence of K12 could strategically reduce the lung and immune capability to release pro-inflammatory cytokines, thus preventing excessive lung inflammation, and the need to proceed to ICU and death	Daily 2 oral BLIS K12 tablets for up to 14 days and placebo	50	https://clinicaltrials.gov/ct2/show/NCT05043376

NCT05175833	Completed	Passo Fundo, Brazil	Effect of oral probiotics *Streptococcus salivarius* K12 and *Lactobacillus brevis* CD2 on the prevention of secondary bacterial pneumonia in patients with severe COVID-19	Phase II randomized clinical trial	*Streptococcus salivarius* K12 combined with *Lactobacillus brevis* CD2 prevent secondary bacterial pneumonia in patients with severe COVID-19	Oral gel containing *Streptococcus salivarius* K12 and *Lactobacillus brevis* CD2 applied in the mouth every 8 hours for 7 days and placebo	70	https://clinicaltrials.gov/ct2/show/NCT05175833
NCT04756466	Active, not recruiting	Santiago De Compo-stela, Spain	Multicenter, randomized, double-blind parallel group pilot study to evaluate the effect of the consumption of a *Lactobacillus* strain on the incidence of COVID-19 in the elderly	Multi-center, randomized, double-blind parallel group pilot study	The administration of a *Lactobacillus* strain improves the immune response in the elderly population, improving the immune response to a possible COVID-19 infection	Experimental group that will receive one capsule with the probiotic strain per day ($3x10^9$ CFU / day) during 3 months	201	https://clinicaltrials.gov/ct2/show/NCT04756466
NCT04420676	Active, not recruiting	Graz, Austria	Synbiotic therapy of gastro-	Randomized, double-blind, placebo	The intake of Omni-Biotic® 10 AAD can	Probiotic mixture (Omni-Biotic®	30	https://clinicaltrials.gov/ct2/s

			intestinal symptoms during COVID-19 infection (SynCov Study)	controlled, telemedicine study	reduce intestinal inflammation and improves dysbiosis in COVID-19 disease	10 AAD) twice a day and placebo		how/NCT04420676
NCT04666116	Recruiting	Sagunto, Spain	Changes in viral load in patients with COVID-19 disease after dietary supplementation with probiotics: a randomized clinical trial	Randomized, single blind clinical trial	Through the administration of specific probiotics, the immune response against the coronavirus COVID-19 could be stimulated, being able to decrease its viral load and secondary symptom-matology	Dietary supplementation in patients with COVID disease admitted to hospital *Dosage not provided*	96	https://clinicaltrials.gov/ct2/show/NCT04666116
NCT04907877	Recruiting	Lviv, Ukraine	Role of nutritional support with probiotics in adult outpatients with symptomatic COVID-19	Randomized Dietary Study	Proposed mixture of *lactobacilli* and *bifidobacteria* facilitate faster recovery from COVID-19 and enhance specific	NordBiotic ImmunoVir, a mixture of *bidido*- and *lactobacteria* administered in a dose of 5 billion once a day for 28	100	https://clinicaltrials.gov/ct2/show/NCT04907877

					immune response to SARS-CoV-2 antigens	days and placebo		
NCT04462627	Recruiting	Brussels, Belgium	Reduction of COVID 19 transmission to health care professionals	Non-Randomized clinical trial	*No related with probiotics administration*	Diagnostic test: blood group determination Diagnostic test: antibody titration Dietary supplement: probiotic *Dosage not provided*	500	https://clinicaltrials.gov/ct2/show/NCT04462627
NCT04813718	Recruiting	Graz, Austria	Post COVID-19 syndrome: a pilot study to explore the gut-lung axis	Randomized clinical trial	Probiotics as a possible modulator of the deranged gut-lung axis in COVID-disease and post-COVID syndrome	Synbiotic: Omni-Biotic Pro Vi 5 and placebo	20	https://clinicaltrials.gov/ct2/show/NCT04813718
NCT04798677	Recruiting	Barcelona, Spain	Efficacy and tolerability of a nutritional supplementation with ABBC-1, a symbiotic combination of beta-glucans	Single-center, randomized, double-blinded, placebo-controlled clinical study	Nutritional supplementation with ABBC1 carry benefits w in volunteers receiving the influenza	Influenza or COVID-19 vaccine followed by 30 days of supplementation with a beta-glucan	90	https://clinicaltrials.gov/ct2/show/NCT04798677

			and selenium and zinc enriched probiotics, in volunteers receiving the influenza or the COVID-19 vaccines		vaccine during autumn 2020 and the COVID-19 vaccine during winter 2021	complex and *Saccharomyces* consortium rich in selenium and zinc and placebo *Dosage not provided*		
NCT05080244	Recruiting	Sherbrooke, Canada	Evaluation of the efficacy of probiotics taken during the acute phase of COVID-19 to reduce the occurrence of long COVID	Randomized clinical trial (double-blinded)	Taking probiotics during the acute phase of the disease reduces the number of patients with long COVID by 25% 90 days after the diagnosis	Probiotics 2 strains 10x109 CFU/capsule (two capsules per day – one closed capsule to swallow and one open capsule mixed with maple syrup for 10 days and one closed capsule per day to swallow for the following 15 days); the treatment will last a maximum of 25 day	618	https://clinicaltrials.gov/ct2/show/NCT05080244
NCT05195151	Recruiting	Sherbrooke, Canada	Modulation of immune responses to	Randomized controlled trial	Taking probiotics one month before	Taking a probiotics capsule	668	https://clinicaltrials.gov/ct2/s

			COVID-19 vaccination by an intervention on the gut microbiota		and one month after the 4th dose of COVID vaccine would result in longer lasting vaccine protection in seniors	containing the probiotics 15 d before and 15 d after booster shot and placebo *Dosage not provided*		how/NCT0519 5151
NCT04941703	Recruiting	Nashville, United States	Investigation of choice alteration of the gut metagenome on COVID-19 severity	Investigator-initiated, single center, blinded, placebo-controlled, randomized clinical trial	Taking oral magnesium citrate and a probiotic will improve the outcome of adults hospitalized with COVID-19	296 ml magnesium citrate + probiotics (2 capsules of probiotics twice daily for six days) and placebo	30	https://clinicalt rials.gov/ct2/s how/NCT0494 1703
NCT04979065	Recruiting	Jakarta, Indonesia	Effect of probiotic and vitamin D supplementati on in modulating gut dysbiosis, nutrition, inflammation and immune status and reduce risk of COVID-19 in	Phase 1: a cross-sectional study with 160 people; Phase 2: a double-blind, randomized, placebo-controlled trial with two arms of intervention	Combination of probiotics and vitamin D supplementati on modulate intestinal dysbiosis, and vitamin D status, in people with overweight and obesity, especially	Combination of two supplement that given separately and placebo *Dosage not provided*	80	https://clinicalt rials.gov/ct2/s how/NCT0497 9065

			obese people: gut-lung axis randomized trial		among frontline health workers			
NCT05195047	Recruiting	Beijing, China	Efficacy and safety of oral *Lactobacillus plantarum* GUANKE (CGMCC NO.21720) in enhancement of antibody level after SARS-CoV-2 vaccination	Prospective, Randomized, Double-blind, Placebo-Controlled Trial (Trial 2)	Oral probiotics in SARS-CoV-2 serum neutralize antibody titer level and T cell response level	1 week of consecutively consuming oral probiotics (1 time/day) followed by SARS-CoV-2 vaccination, then 2 weeks of consecutively consuming oral placebo (1 time/day)	44	https://clinicaltrials.gov/ct2/show/NCT05195047
NCT05227170	Recruiting	Milwaukee, United States	Impact of *Lp*299v on vascular function in patients with PASC	Double-blind, randomized, placebo-controlled clinical trial	Supplementation with *Lp*299v will attenuate SARS-CoV-2 associated endothelial dysfunction by reducing cf-mtDNA, TLR9 activation, and inflammation	*Lactobacillus plantarum* 299v in fermented oat drink (20 billion colony forming units once daily for 8 weeks) and placebo	80	https://clinicaltrials.gov/ct2/show/NCT05227170
NCT04877704	Not yet recruiting	London, United Kingdom	The effect of Symprove, a multi-strain	Randomized clinical trial with single	Taking the probiotic Symprove	Probiotic Symprove /	60	https://clinicaltrials.gov/ct2/s

			probiotic, as an adjuvant in the management of COVID-19 in hospitalized patients	group assignment	reduces the length of hospital stay of patients with symptomatic respiratory COVID-19 infection	placebo daily for 3 months *Dosage not provided*		how/NCT04877704

References

1. Tang, H.; Bohannon, L.; Lew, M.; Jensen, D.; Jung, S.-H.; Zhao, A.; Sung, A. D.; Wischmeyer, P. E. Randomised, Double-Blind, Placebo-Controlled Trial of Probiotics To Eliminate COVID-19 Transmission in Exposed Household Contacts (PROTECT-EHC): A Clinical Trial Protocol. BMJ Open 2021, 11 (5), e047069. https://doi.org/10.1136/bmjopen-2020-047069.
